# 

**Published:** 1900-10

**Authors:** 


### Society Notes.

### Tri-State Medical Society of Alabama, Georgia and Tennessee.

*Dear Doctor:*

The twelfth annual meeting of the Tri-State Medical Society will meet at Chattanooga, Thursday, Friday and Saturday, October 11, 12 and 13, 1900, just after the meeting of the Mississippi Valley Medical Association, Asheville, N. C. A rate of one fare for the round trip will be in force on the occasion of the reunion of the Army of the Cumberland and the Spanish-American War Veterans.

The committee in charge would be pleased to have you attend and read a paper.

Kindly send title to the secretary at an early date for the preliminary program soon to be issued.

Fraternally yours,

FRANK TRESTER SMITH, Secretary,
Chattanooga, Tenn.

### Southern Surgical and Gynecological Association.

The meeting of the Southern Surgical and Gynecological Association will be held in Atlanta, November 13, 14 and 15, under the presidency of Dr. A. M. Cartledge, of Louisville. Prospects are splendid for successful session. Members of the medical profession are cordially invited to attend.

